# Study on the Relationship between Urban Street-Greenery Rate and Land Surface Temperature Considering Local Climate Zone

**DOI:** 10.3390/ijerph20043294

**Published:** 2023-02-13

**Authors:** Xinyue Wang, Zhengrui Li, Shuangxin Ding, Xiufeng Sun, Hua Qin, Jianwan Ji, Rui Zhang

**Affiliations:** 1 College of Horticulture and Landscape Architecture, Southwest University, Chongqing 400700, China; 2 China Merchants Group, China Merchants Shekou Chengdu Region, Chengdu 610000, China; 3 School of Geography Science and Geomatics Engineering, Suzhou University of Science and Technology, Suzhou 215009, China; 4 Northwest Institute of Eco-Environment and Resources, Chinese Academy of Sciences, Lanzhou 730000, China

**Keywords:** street-greenery rate, land surface temperature, correlation analysis, local climate zone, Chongqing

## Abstract

Relationship exploration between the street-greenery rate (SGR) of different street types and land surface temperature (LST) is of great significance for realizing regional sustainable development goals. Given the lack of consideration of the local climate zone concept (LCZ), Chongqing’s Inner Ring region was selected as a case to assess the relationship between SGR and LST. Firstly, the LST was retrieved based on Landsat 8 imagery, which was calibrated by the atmospheric correction method; next, the street-greenery rates of different streets were calculated based on the semantic segmentation method; finally, street types were classified in detail by introducing LCZ, and the relationship between SGR and LST was investigated. The results showed that: (1) The LST spatial distribution pattern was closely related to human activity, with the high-temperature zones mainly concentrated in the core commercial areas, dense residential areas, and industrial cluster areas; (2) The average SGR values of expressways, main trunk roads, secondary trunk roads, and branch roads were 21.70%, 22.40%, 24.60%, and 26.70%, respectively. The level of SGR will decrease when the street width increases; (3) There is a negative correlation between the SGR and the LST in most streets. Among them, the LST of secondary trunk roads in low-rise and low-density built-up areas with a south-north orientation had a strong negative correlation with the SGR. Moreover, the wider the street, the higher the cooling efficiency of plants. Specifically, the LST of streets in low-rise and low-density built-up areas with south-north orientation may decrease by 1°C when the street-greenery rate is increased by 3.57%.

## 1. Introduction

Since the implementation of the reform and opening-up policy in 1978, the urbanization rate of China has gradually increased [1]. According to the *China Statistical Yearbook* (2022), published by the National Bureau of Statistics, the proportion of China’s urban population increased from 17.92% in 1978 to 64.72% in 2021, with an average annual growth rate of 1.09% [2]. Based on a United Nations report, the proportion of the world’s population living in urban areas is expected to increase to 68% by 2050 [3]. However, along with the rapid development of urban construction and the urban economy, a large number of natural surfaces have been replaced by impervious surfaces [4,5], causing a series of urban thermal environment problems. Among them, the urban heat island effect is one of the apparent problems affecting urban residents’ quality of life [6,7,8,9].

The urban heat island effect refers to the phenomenon whereby the land surface temperature (LST) in the urban center areas is higher than that in the suburbs due to the massive accumulation of heat caused by human activity [10,11]. Urban streets are one of the components of urban outdoor public space, and they refer to linear roads enclosed by buildings on both sides [12]. As part of the impervious surface, the urban streets have the features of fast heat absorption and small heat capacity. When urban streets absorb a significant amount of solar radiation, their temperature will rise rapidly, further intensifying the urban heat island effect [13]. Hence, it is of great significance to improve the thermal environment of urban streets. To date, in the multi-scale studies of the urban heat island effect, it was found that urban green infrastructures, such as street greening, can effectively mitigate the urban heat island effect [14]. To be specific, Huang et al. summarized the quantitative indicators and found that the reasonable block size should be controlled within 200 m based on statistics and comparative analysis in dozens of large cities abroad [15]. In addition, he suggested that this block size should be further controlled within 150–200 m after combining it with the domestic urban development state [15]. Jin studied the block thermal environment influencing factors by establishing a numerical simulation model [16]. He found that different materials of the underlying surface would affect the outdoor thermal environment, and suggested that dark materials should be avoided when building streets [16]. Du took Guizhou city as the study area, and found that the orientation, height-width ratio, greening, and surface reflectivity will exert an influence on regional thermal environment. Among them, at noon, the street greening will exert significant influence on the thermal environment of streets [17].

With the exception of street greening, existing studies have found that the geometric shape of urban streets could also have a significant impact on a street’s thermal environment [18]. Specifically, building differences, street orientation, and other factors could affect the heat exchange process between the street surface and the surrounding environment to varying degrees [18]. In addition, the degree of influence is determined by building height, building density, sky visibility factor, street height-width ratio, and street orientation [19,20]. For example, Cao found that the street surface temperature would decrease with the increase in street width [21]. On the other hand, he found that street wind direction and street width would also have a particular impact on the thermal environment of a street [21]. Ali-Toudert et al. also found that a street’s geometric shape would affect the street thermal environment to a certain extent. Among them, the street sky visibility factor was positively correlated with a street’s thermal environment. In addition, for streets with same sky visibility factor, the orientation would also have a significant impact a street’s thermal temperature. Specifically, streets with the east-west orientation had the worst thermal environment [22]. Bourbia discussed the impact of the size of a street and the open space proportion on a street’s thermal environment [23]. In addition, the road centrality was also related with socio-demographic parameters [24]. It can generally be concluded that a street’s LST was greatly affected by street geometry, including the street height-width ratio, sky visibility factor, and street orientation.

The traditional urban street greening monitoring method is the vegetation coverage calculation based on satellite remote sensing images. However, this method has obvious shortcomings. It is difficult to get the vertical distribution information of plants. In recent years, research on street greening has begun to monitor plants from the perspective of pedestrians. Baidu Street View images are used to obtain the distribution of street greening through in-depth computer learning and are applied to various urban studies.

The street-greenery rate (SGR) refers to the proportion of green plants in the whole field of vision seen from the human perspective. The proposal of the concept of SGR has provided a new evaluation standard for road greening quality, and a new idea for urban road greening design, which better reflects the people-oriented design concept [25]. When describing the greening level of urban streets, except for the traditional green space rate, green coverage rate and other indicators, the average SGR value of multiple sample points of the streets can also be used to reflect the greening level [26]. Feng et al. analyzed the feasibility of extracting the street green vision rate from Baidu Street View pictures [27]. The research results showed that the street SGR had a significant positive correlation with the vegetation coverage. This study proved that it was feasible to take the urban SGR as an evaluation index to reflect the street greening level [27]. Xu et al. also used Baidu Street View pictures to calculate the street SGR in 2020, and the research results indicated that the influencing factors of SGR included the plant morphology index, plant configuration form, street width and street three-dimensional greening degree [28]. 

Based on the aforementioned analysis, existing studies mainly focused on the study of the influence of different street geometry and street greening on streets’ LST. However, there also existed some limitations: (1) Few studies reported the relationship between the influence of geometric shape and greening on surface temperature; and (2) Few studies introduced the local climate zone (LCZ) concept to analyze the relationship of SGR and land surface temperature (LST) at different LCZs. Hence, this study aimed to answer the question: what is the relationship between SGR and LST on streets of different types, different street orientations, and different LCZs? Specifically, compared with existing studies, this study could provide detailed information on the relationship of streets’ SGR and LST. Firstly, LST was retrieved based on Landsat 8 remote sensing imagery; next, SGR was calculated by image semantic segmentation technology based on Baidu Street View pictures; finally, LCZ was introduced to further classify the streets and analyze the relationship between LST and SGR at different street types. This paper is organized as follows: Section 2 describes the study area and methods, which include the datasets acquisition and the calculation of SGR; Section 3 presents the SGR accuracy, the statistical and spatial distribution results, and the relationship between SGR and LST at different LCZs and different street types; Section 4 validates the relationship between SGR and LST, the accuracy of SGR, and presents the implications and limitations of this study; and Section 5 presents the main findings of this paper.

## 2. Materials and Methods

### 2.1. Study Area

**Figure 1:**
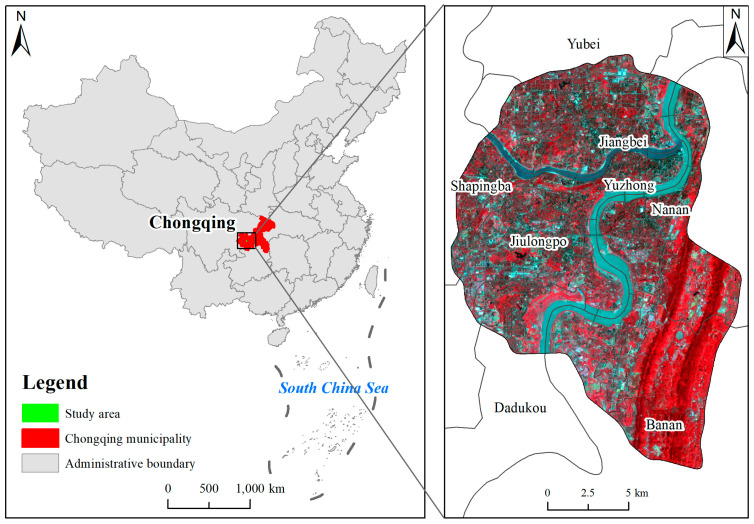
Location of the study area.

Chongqing is located in the southwest of China and at the upper reaches of the Yangtze River (Figure 1). Based on the actual range of Chongqing’s urban built-up area and the planning of Chongqing’s traffic network, this study selected the metropolitan built-up area bounded by Chongqing Inner Ring Expressway as the research area; it covers the whole area of the Yuzhong District, as well as part of the seven main urban areas of Jiangbei, Yubei, Nanan, Banan, Shapingba, Jiulongpo, and Dadukou, with a total area of about 296 km^2^. Chongqing belongs to the subtropical monsoon area. The total annual sunshine hours are 1000–1200 h, the annual average temperature is about 18 °C, the minimum temperature in winter is about 6–8 °C, and the summer is hot. In addition, a southeast wind prevails, and the maximum temperature is about 35 °C.

### 2.2. Data Sources

In this study, the data sources included remote sensing datasets, administrative boundary data of the Chongqing urban built-up area, road network data of the Chongqing metropolitan built-up area, meteorological data at the satellite transit moment, and Baidu Street View pictures. 

Specifically, remote sensing datasets included the Landsat 8 imagery, downloaded from the Geospatial Data Cloud (https://www.gscloud.cn/) (accessed on 15 March 2021), which was chosen to retrieve LST, with a spatial resolution of 100 m [29]. The shooting time of this image was 03:27:21 (GMT) on 20 August 2019, with the path and row of 128 and 39, respectively. 

The meteorological data were provided by the Chongqing Meteorological Service (http://cq.cma.gov.cn/) (accessed on 15 March 2021) at 03:30:00 (GMT) on 20 August 2019, which was used for the accuracy evaluation of the LST retrieval result. 

The administrative boundary of the Chongqing urban built-up area was downloaded from the National Geomatics Center of China, which was produced in 2021 (http://www.ngcc.cn/ngcc/html/1/index.html) (accessed on 30 May 2021) and used for data clipping and mapping. 

According to the Landsat 8 imagery in 2019, land cover data was produced based on the supervised classification method in ENVI 5.6 software.

Road network data was downloaded from the Open Street Map website (https://www.openstreetmap.org/#map=11/22.3567/114.1363) (accessed on 21 March 2021). Chongqing’s urban spatial form data and Baidu Street View pictures were acquired from Baidu map using Python. Specifically, Chongqing’s urban spatial form data included the latitude and longitude coordinates, height, and vector of buildings in 2019. 

**Figure 2:**
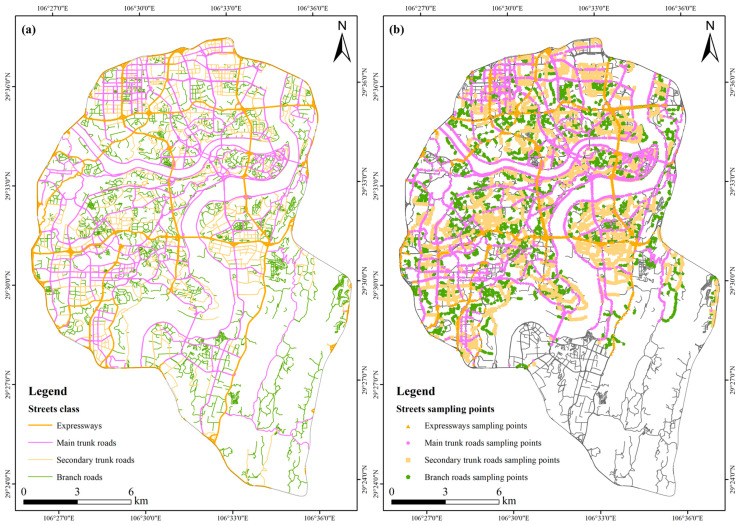
Spatial distribution map of streets in the study area (**a**), street sampling points in the study area (**b**).

To get consistent locations of these datasets, firstly, locations of different street types were calibrated based on images from Google Earth images in 2019, and the software was downloaded from the 91weitu website (https://www.91wemap.com/) (accessed on 18 May 2021). The Google Earth images had a standard projection system with WGS_1984; however, the coordinates of the Baidu map had a slight deviation. Hence, Google Earth was set as a reference map to calibrate other datasets acquired from the Baidu map. Then, based on spatial analysis tools in ArcGIS 10.8 software, sampling points (a total of 15,862) of streetscape images every 100 m along the street line were generated, and the longitude and latitude coordinates of each sampling point were calculated; finally, Python was used to download Baidu Street View pictures from four directions of each sampling point (Figure 2). Except for the area not covered by Baidu Street View, a total of 60,586 Baidu Street View pictures were obtained. In this study, these extracted Baidu Street View pictures were acquired from June to August of 2019, which means that we could ignore the impact of the seasonal changes of plants on SGR. 

### 2.3. Methods

The research methodology of this paper consisted of four main parts, which included the following: (1) the retrieval of LST; (2) the calculation of SGR; (3) the classification of street types; and (4) a correlation analysis. Section 2.3.1, Section 2.3.2, Section 2.3.3 and Section 2.3.4 present the calculation formula in detail.

#### 2.3.1. Retrieval of LST

In the study of the urban heat islands effect, the monitoring of LST is one of the main methods to understand the spatial and temporal distribution characteristics of a heat island, and the LST retrieval based on satellite remote sensing images is the primary method. In recent years, satellite remote sensing images, including Landsat series, ASTER, MODIS, and other products, were used to retrieve LST [30]. Among them, Landsat 8 images launched in 2013 have the advantages of high spatial resolution, rich spectral information, and long-term monitoring. Hence, Landsat 8 images have been widely used [31,32]. To date, the single band algorithm and window splitting algorithm were the two main types in retrieving LST [33,34]. Specifically, the single band algorithm included the atmospheric correction method, single channel algorithm, image-based algorithm, and single window algorithm [35,36,37,38]. Among them, the atmospheric correction method, also named the radiative transfer equation method, is a traditional algorithm based on the atmospheric radiative transfer model. Its advantage lies in that it could be applied to any thermal infrared remote sensing band [39]. Equations (1)–(8) are calculation formulas.

(1) $$L_{\lambda }=\left[\varepsilon B\left(T_{s}\right)+\left(1-\varepsilon \right)L_{down}\right]\tau +L_{up}$$

(2) $$B\left(T_{s}\right)=\frac{L_{\lambda }-L_{up}-\tau \left(1-\varepsilon \right)L_{down}}{\tau \varepsilon }$$

(3) $$T_{s}=\frac{K_{2}}{\ln\left(\frac{K_{1}}{B\left(T_{s}\right)}+1\right)}$$

(4) $$\varepsilon =0.004P_{v}+0.986$$

(5) $$P_{v}=\left[\frac{\left(NDVI-NDVI_{soil}\right)}{\left(NDVI_{veg}-NDVI_{soil}\right)}\right]$$

(6) $$\varepsilon_{water}=0.995$$

(7) $$\varepsilon_{surface}=0.9625+0.0614\times P_{v}-0.0461\times P_{v}^{2}$$

(8) $$\varepsilon_{building}=0.9589+0.086\times P_{v}-0.0671\times P_{v}^{2}$$

where $L_{\lambda }$ denotes the thermal infrared radiation brightness value received by satellite sensor; $L_{up}$ indicates the upwelling radiance of atmosphere; $L_{down}$ represents the downwelling radiance of atmosphere; ε is the surface emissivity; τ is the atmospheric transmission at thermal infrared band; $L_{up}$, $L_{down}$ and τ are acquired from NASA website (http://atmcorr.gsfc.nasa.gov/) (accessed on 23 August 2021) after inputting the time, latitude and longitude. In this study, their values equal 4.77 $W/\left(m^{2}\times \mu m\times \mathrm{sr}\right)$, 7.06 $W/\left(m^{2}\times \mu m\times \mathrm{sr}\right)$, and 0.44, respectively. $T_{s}$ is the actual surface temperature; $B\left(T_{s}\right)$ is the radiance brightness of the black body at temperature $T_{s}$; *K*_1_ and *K*_2_ are constant values with the value of 774.89 $W/\left(m^{2}\times \mu m\times \mathrm{sr}\right)$ and 1321.08 *K*, which are provided by the metadata file of the Landsat 8 image; $P_{v}$ represents the vegetation coverage, which is calculated based on NDVI index; $NDVI_{veg}$ and $NDVI_{soil}$ represent the NDVI value of pure vegetation pixels and bare soil or no vegetation coverage pixels. In this study, the $NDVI_{veg}$ and $NDVI_{soil}$ values are 0.70 and 0.05, respectively [40,41]; When the NDVI value at certain pixel is greater than 0.70, the value of $P_{v}$ equals 1.00, while the NDVI value at a certain pixel is less than 0.05, the value of $P_{v}$ equals 0.00. $\varepsilon_{water}$, $\varepsilon_{surface}$ and $\varepsilon_{building}$ represent the surface emissivity of the water pixel, natural surface pixel, and urban area pixel, respectively.

#### 2.3.2. Calculation of SGR

**Figure 3:**
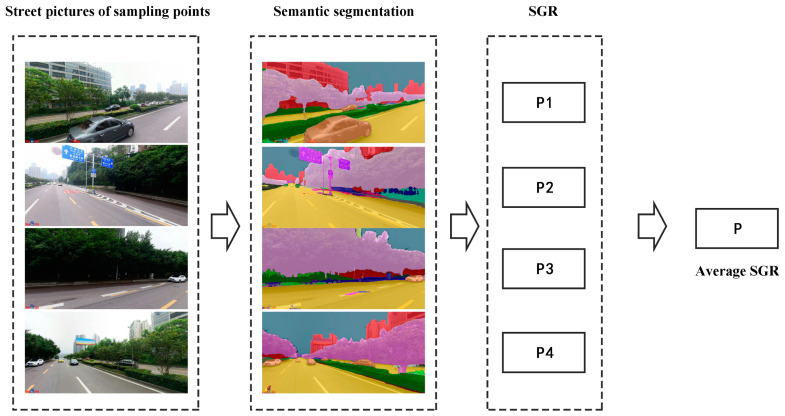
SGR calculation steps of sampling points.

Based on Baidu Street View pictures, this study used the image semantic segmentation method to calculate each street’s SGR from four directions [42,43,44]. It included six steps, which were (1) the acquisition of the city street network; (2) the sampling points’ generation of street scene images per 100 m in street network; (3) the acquisition of Baidu Street View pictures of sampling points from four directions; (4) the semantic segmentation of Baidu Street View pictures based on a full convolution network; (5) the percentage calculation of vegetation pixels in each picture; and (6) the average value of the street-greenery rate from four directions. Figure 3 displays the calculation method of the SGR of the sampling point.

#### 2.3.3. Classification of Street Types

A street’s thermal environment is affected by the climate environment of the surrounding areas. First, there is a significant correlation between the surface temperature and the density of urban impervious and underlying surfaces [45,46]; secondly, the spatial distribution of buildings in horizontal and vertical directions is extremely uneven. The buildings and narrow streets formed by the buildings will weaken the effect of heat diffusion, thus affecting the surface temperature [47]; thirdly, factors including the height-width ratio of a street, sky visibility, and building density are often used as the basis for the division of a local climate zone. 

Hence, in this study, in the division of different street types, the concept of LCZ was introduced first, and the urban areas where the street was located were preliminarily divided by referring to the LCZ division method. The street types were then further distinguished according to the direction and street width. Specifically, LCZ was promoted by Stewart and Oke in 2012, and it referred to the area with the same surface coverage, similar spatial form, building materials, and similar human activities within the range of several hundred meters to several kilometers in one city [48,49]. Based on the concept of LCZ, it could be divided into 17 types [48]. Combined with the actual situation of the study area, the LCZ could be divided into ten types (Table 1). 

Combined with the specific range of impervious surface fraction and pervious surface fraction proposed by Mills [49], the parameter range of block division is shown in Table 2.

#### 2.3.4. Correlation Analysis

A correlation analysis is a statistical analysis method that is used to study the correlation between two or more random variables in the same position [50,51]. Equation (9) is the calculation formula.

(9) $$R_{xy}=\frac{\sum_{i=1}^{n}\sum_{i=1}^{n}\left(x_{i}-\bar{x}\right)\left(y_{i}-\bar{y}\right)}{\sqrt{\sum_{i=1}^{n}\sum_{i=1}^{n}{\left(x_{i}-\bar{x}\right)}^{2}\sum_{i=1}^{n}\sum_{i=1}^{n}{\left(y_{i}-\bar{y}\right)}^{2}}}$$

where $R_{xy}$ denotes the correlation coefficient between x and y; $x_{i}$ and $y_{i}$ are the LST and SGR at *i*_th_ point; and $\bar{x}$ and $\bar{y}$ are the average value of LST and SGR.

## 3. Results

### 3.1. Spatial Characteristic of LST Result

**Figure 4:**
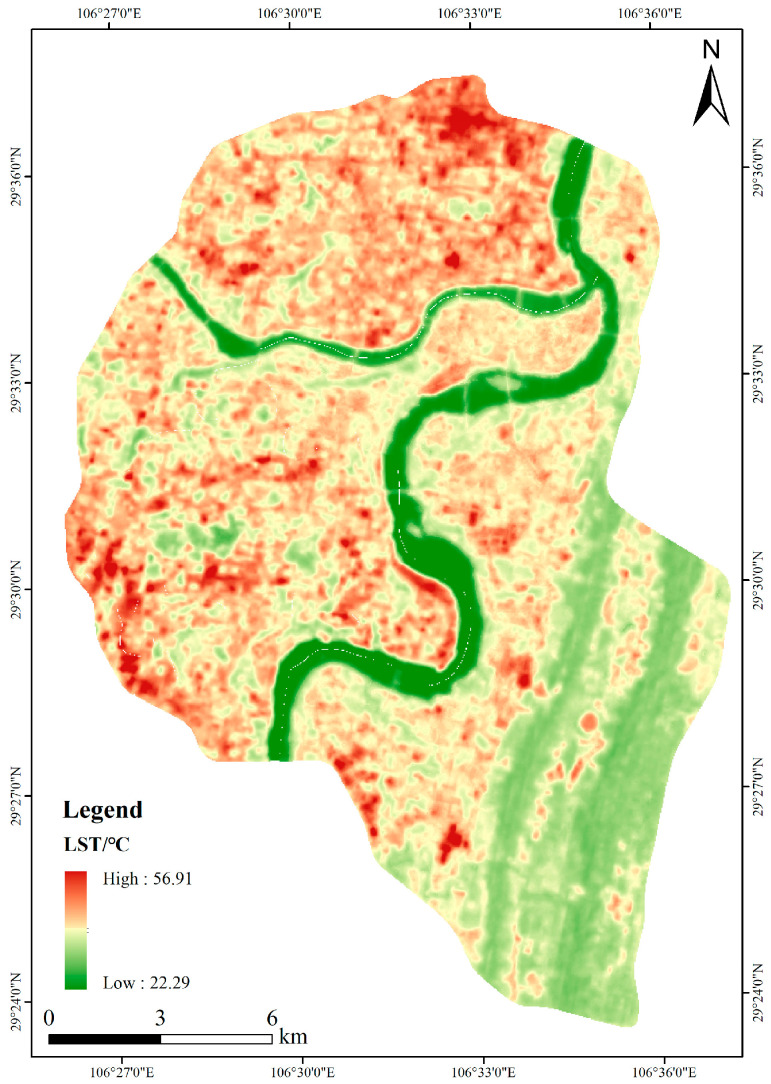
LST result based on the atmospheric correction method.

**Figure 5:**
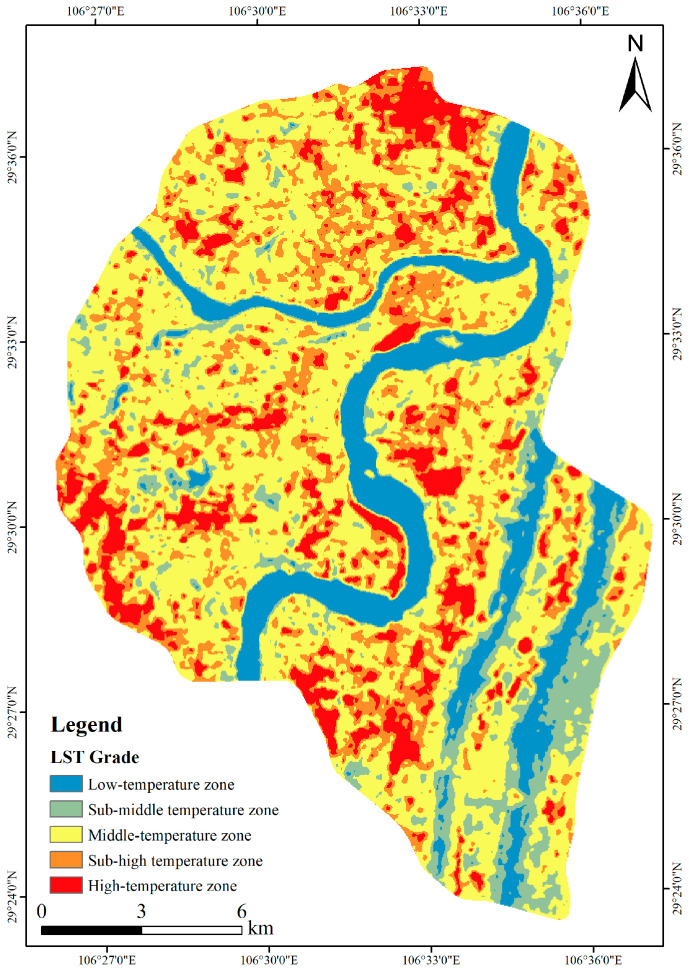
Different LST grades.

Based on the atmospheric correction method, the LST spatial distribution map was acquired on 20 August 2019 (Figure 4). In addition, combined with the application of the mean standard deviation division method in the previous study [42,52], this study used this method to divide the LST of the study area into five grades, which were high-temperature zone, sub-high temperature zone, middle-temperature zone, sub-middle temperature zone, and low-temperature zone (Figure 5).

According to Figure 4 and Figure 5, the high-temperature zones were mainly concentrated in the core commercial areas, dense residential areas, and industrial cluster areas. The LST spatial distribution patterns were closely related to human activities. Factors such as building height and surface materials would also affect the LST spatial distribution patterns. Reasonable urban planning, green space construction and other methods could effectively mitigate the heat island effect. However, for these built-up areas in the urban center, it was challenging to reserve large land areas for green space construction.

### 3.2. Spatial Distribution Map of Streets LST

**Figure 6:**
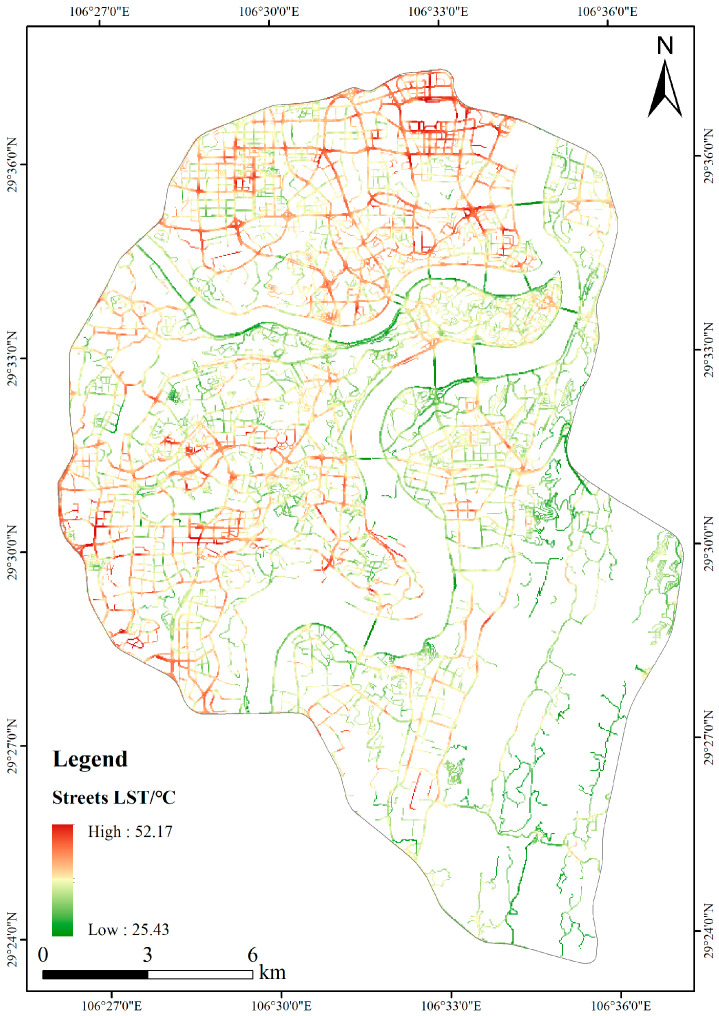
Spatial distribution map of streets LST.

Based on the calibrated street network data, the streets’ LST were extracted (Figure 6).

According to Figure 6, the streets with a high LST were mainly concentrated in the northern and western regions. The most severe regions with high temperatures were mostly around Chongqing North Railway Station. Based on satellite images, buildings in these regions were distributed densely, with many commercial and residential buildings. Streets with high LST in western regions were mainly located in Mawangchang and Hualong in the Jiulongpo District. Except for many residential buildings in these regions, there also existed many building material markets. The buildings in these markets mainly belonged to low-rise and large-area buildings, accompanied by a large area of hard paved ground. Streets with mid-level temperatures were primarily distributed in the Yuzhong Peninsula, the Nanping Business District, and the Shapingba Business District. These regions had dense buildings on both sides of the streets. Streets in these regions mainly belonged to the urban secondary trunk roads and branch roads. The shading effect of buildings on these streets was more evident than that of streets in high LST. Streets with low-temperature surfaces were mainly distributed on both sides of the river and in the eastern and southern regions. By comparing these streets’ LST on both sides of the river, it could be seen that these streets’ LST on the south bank of the river were significantly lower than those on the north bank of the river. This is because the rivers flowing through the city would reduce the air temperature of the surrounding environment, thus increasing the heat exchange between the surface and the air of the streets along the river and reducing street surface temperature. On the other hand, these north bank riverside streets absorbed a significant amount of solar radiation, causing their LSTs to be higher than those of the south bank riverside streets. The eastern and southern Chongqing belonged to mountainous areas (Nanshan), with high green coverage and low building density. Plant shading and transpiration affected the total amount of solar radiation entering these streets; hence, the air temperature on these streets was lower. Therefore, these streets’ LST were significantly lower than in other areas.

**Figure 7:**
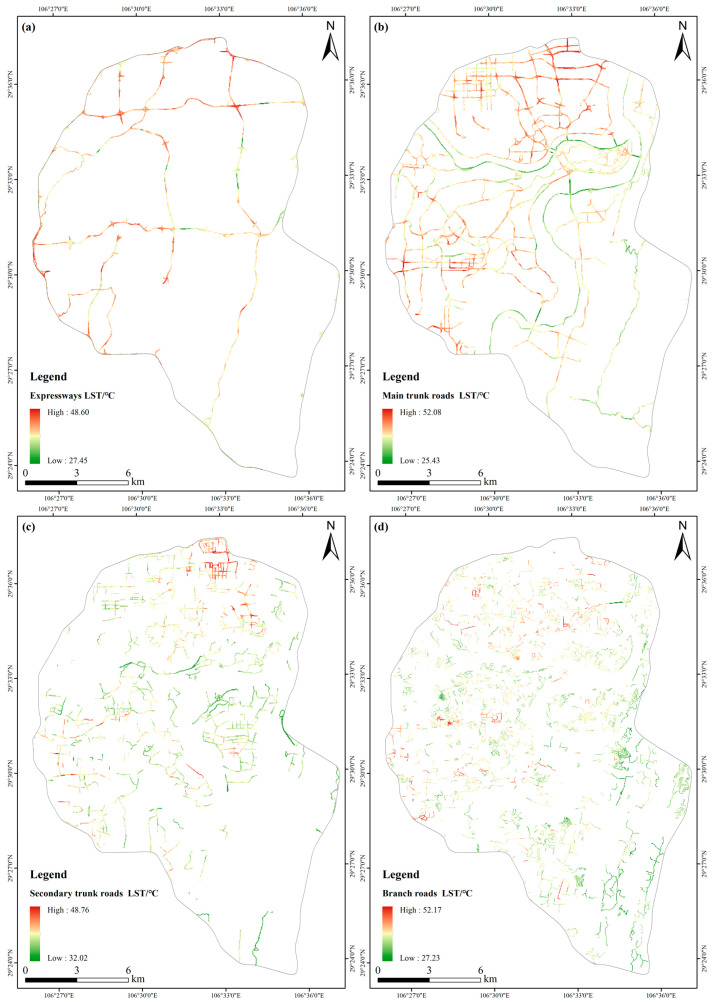
Spatial distribution map of streets’ LST of different types: (**a**) Expressways; (**b**) Main trunk roads; (**c**) Secondary trunk roads; (**d**) Branch roads.

To further understand these streets’ LSTs of different types, the streets in the study area were divided into expressways, main trunk roads, secondary trunk roads, and branch roads (Figure 7).

According to Figure 7, the streets’ LST of expressways and main trunk roads were higher than the secondary trunk roads and branch roads. In addition, these streets’ width also exerted a specific influence on LST. Furthermore, the expressways and main trunk roads with high LST were mainly distributed in those streets with an east-west orientation, indicating that street orientation will affect the street LST.

### 3.3. Spatial Distribution of SGR

**Figure 8:**
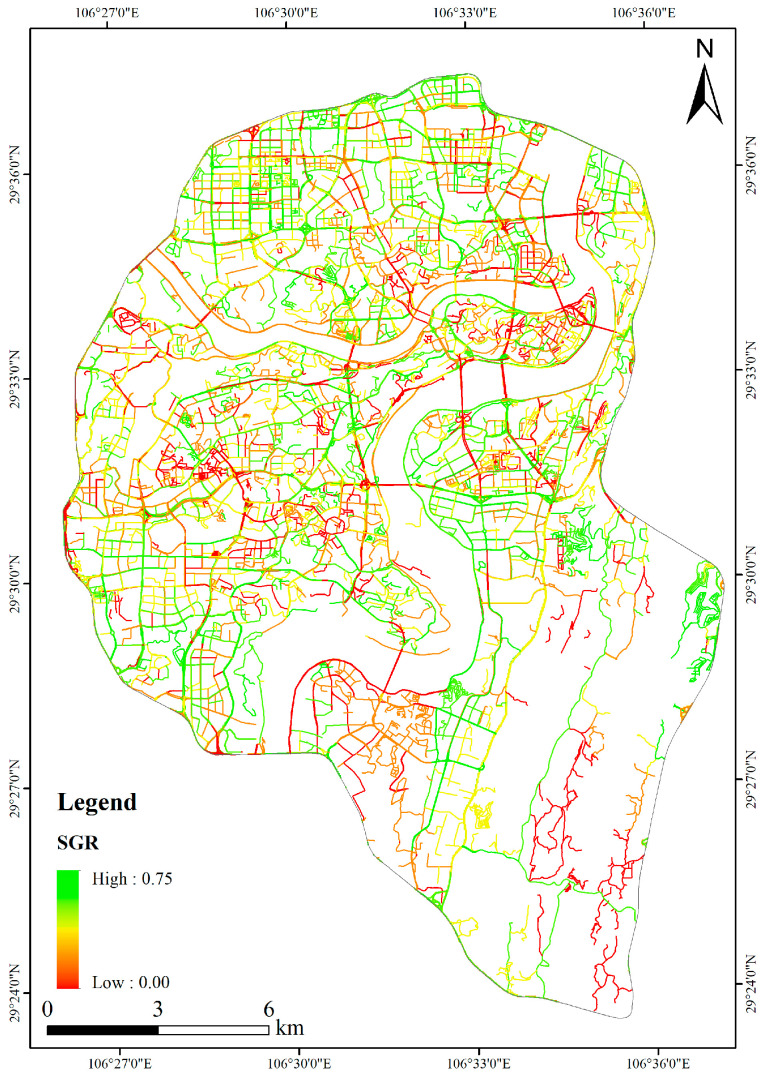
Spatial distribution map of SGR.

Figure 8 shows the spatial distribution of SGR in the study area.

According to Figure 8, the average value of the expressways’ SGR was 21.70%, and the average value of the main trunk roads’ SGR was 22.40%. As for the secondary trunk roads and branch roads, their average SGR values were 24.60% and 26.70%, respectively. Generally, the streets’ width will decrease the level of SGR. This is because the increase in a street’s width will lead to a more significant coverage of the field of view, and the proportion of plants in the field of view will decrease.

### 3.4. Spatial Distribution of Different Street Types

**Figure 9:**
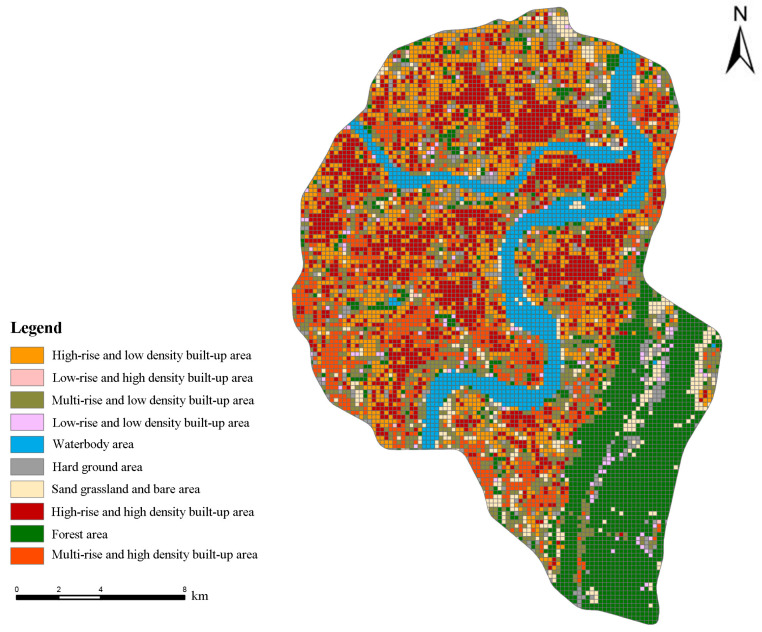
Spatial distribution of different street types.

Figure 9 shows the spatial distribution of varying street types in the study area.

According to Figure 9, forest areas were mainly distributed in the southeastern regions. Waterbody areas were primarily located in the central regions. High-rise and low-density built-up areas, and high-rise and high-density built-up areas were dominant regions. Among them, high-rise and high-density built-up areas were mainly found along the waterbody areas. For sand grassland and bare areas, they were sporadically distributed in the southeastern regions. Generally, in the entire study area, high-rise built-up areas accounted for the highest percentage of all built-up types, followed by multi-rise areas. However, low-rise built-up areas accounted for the low rate.

## 4. Discussion

### 4.1. Validation of SGR Calculation Results

**Figure 10:**
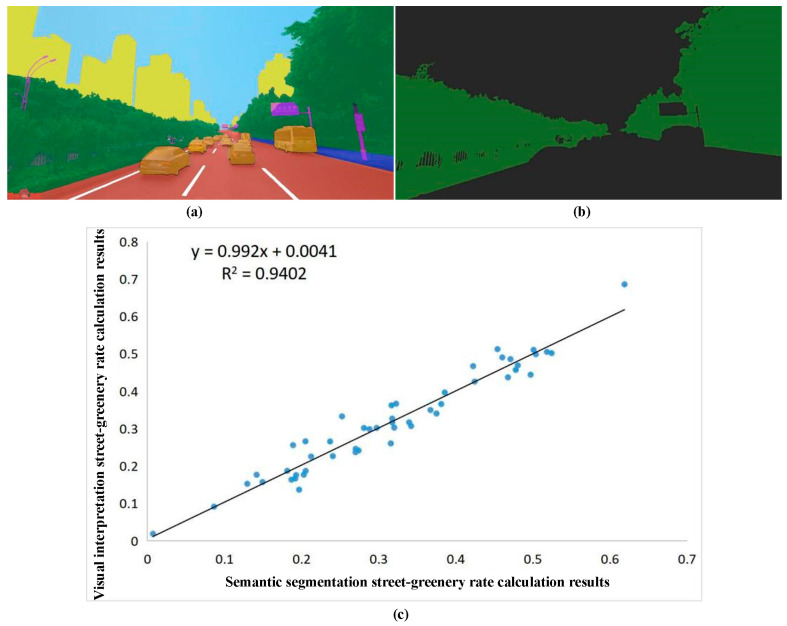
Comparison of two SGR calculation methods: (**a**) Semantic segmentation result; (**b**) Visual interpretation result; (**c**) Scatter plot between two methods.

To validate the effectiveness of SGR calculation results, fifty Baidu Street View pictures were randomly selected to compare their SGR based on semantic segmentation and visual interpretation methods. Specifically, semantic segmentation methods based on the full convolution network were applied to all Baidu Street View pictures automatically, and the street-greenery rate of each picture was then calculated. For visual interpretation methods, these fifty selected pictures were digitalized, their SGR was calculated. Finally, a scatter plot was drawn between both methods (Figure 10).

The key in calculating SGR was the accurate detection of plants. According to Figure 10, both methods showed relatively good detection results, indicating that both methods displayed good consistency. However, there existed slight differences between the two methods. The shadow of plants may be recognized as other types based on the semantic segmentation method. However, objects in shadow areas could be accurately detected based on a visual interpretation. Generally, the SGR calculation result when the semantic segmentation method was used was accurate, with an R^2^ of 0.94.

**Figure 11:**
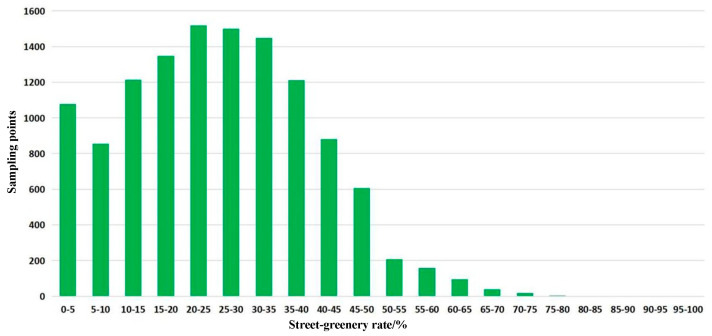
Statistical results of sampling points of the street-greenery rate.

To further validate the SGR results, the statistical results of SGR were calculated (Figure 11). The average SGR of the study area was 24.30%, which was higher than the global average value (19.03%) [53]. Based on the grading standard [28], this study divided the SGR into five grades, which were poor (0.0–5.0%), fair (5.0–15.0%), moderate (15.0–25.0%), good (25.0–35.0%), and excellent (≥35.0%). Combined with grading results, the proportion of streets with poor greening levels in the study area was 8.85%, the proportion of streets with fair greening levels was 16.97%, the proportion of streets with a moderate greening level was 23.53%, the proportion of streets with a good greening level was 24.21%, and the proportion of streets with an excellent greening level was 26.43%. The reasons for these streets having poor greening levels were because of the particular terrain. Chongqing is a mountainous city. The height difference in urban built-up regions varies greatly, which led to a higher proportion of tunnels and viaducts in the urban traffic system compared with more normal cities. In addition, the available space on both sides was less, which did not meet the requirements for street greening construction, thus leading to an increase in the proportion of low-green visibility streets within the study area.

### 4.2. Relationship Exploration between Streets’ SGR and LST

Based on the block division rule, the streets in different blocks were further divided according to street orientation and width. The streets could be divided into four types based on their width, which were expressways, main trunk roads, secondary trunk roads, and branch roads. In different block types, street orientation and street class were also taken into consideration. Finally, seventy-two street types were acquired. Table 3 displays the correlation coefficient between the streets’ SGR and LST at different types.

Given the influence sampling points, the correlation of these street types was not calculated with sampling points less than 50. Based on Table 3, there was a negative correlation between the SGR and the LST in most streets, but the correlation results in different regions were not the same. On the other hand, these street types with high correlation were the south-north expressways, main trunk roads, secondary trunk roads, and branch roads in multi-rise and low-density built-up areas. Among these four types of streets, street greening had the best cooling effect on their surface temperature.

Due to insufficient sampling points of low-rise built-up buildings in the study area, the correlation between streets’ SGR and LST was not calculated. Therefore, the comparative analysis was mainly conducted between multi-rise and high-rise built-up areas. Generally, whether the street orientation was south-north or east-west, the correlation coefficient in low-rise built-up areas was higher than in high-rise built-up areas. The buildings in low-density built-up areas provided less shading area for street space, and the blocking of solar radiation depended more on the shading effect of greening. Hence, the correlation between SGR and LST in multi-rise and low-density built-up areas was higher. Comparing streets with different street orientations in multi-rise and low-density built-up areas, the correlation between the streets’ SGR and LST in the south-north orientation was higher than in the east-west orientation. This was mainly because the shading area buildings of these streets with an east-west orientation could provide more shade. In addition, influenced by the prevailing southeast wind in summer, streets with a south-north orientation were more conducive to airflow. To these streets with a south-north orientation, the correlation between the streets’ SGR and LST in multi-rise and low-density built-up areas was higher than in high-rise and low-density built-up areas. This was mainly because the high-rise buildings could block more solar radiation compared with multi-rise buildings. Generally, the lower the height and density of buildings, the better the cooling effect of street greening. At the same time, the cooling effect of these streets with a south-north orientation was better than those with an east-west orientation. Therefore, in these streets, the LST could be reduced by increasing the SGR.

**Figure 12:**
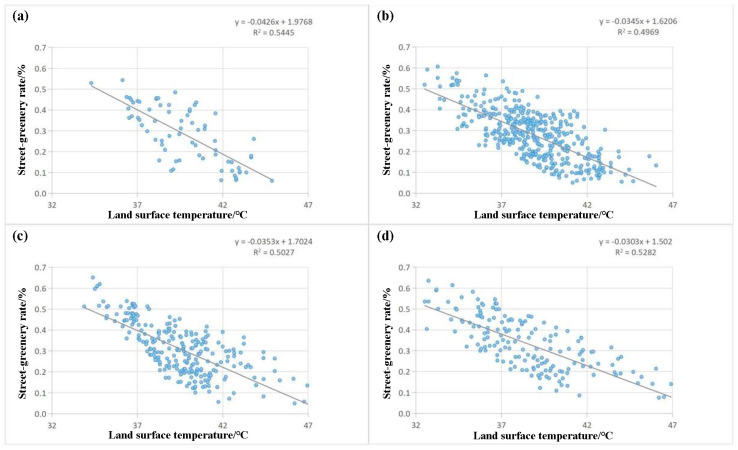
Relationship between the streets SGR and LST of different types in multi-rise and low-density built-up areas, (**a**) Expressways; (**b**) Main trunk roads; (**c**) Secondary trunk roads; (**d**) Branch roads.

For these streets in low-rise and low-density built-up areas with a south-north orientation, fitting formulas of different road types were drawn (Figure 12).

Based on Figure 12, the fitting formula of expressways was *y* = −0.0426*x* + 1.9768, and R^2^ equaled 0.5445. According to this linear modeling result, the LST of this street type may be decreased by 1 °C when the SGR increased by 4.26%. The fitting formula of the main trunk road was *y* = −0.0345*x* + 1.6206, and R^2^ equaled 0.4969. According to this linear modeling result, the LST of this street type may be decreased by 1 °C when the SGR increased by 3.45%; The fitting formula of the secondary trunk roads was *y* = −0.0353*x* + 1.7024, R^2^ equaled 0.5027. According to this linear modeling result, the LST of this street type decreased by 1 °C when the SGR increased by 3.53%. The fitting formula of a branch road was *y* = −0.0303*x* + 1.5020, and R^2^ equaled 0.5282. According to this linear modeling result, the LST of this street type decreased by 1 °C when the SGR increased by 3.03%. Generally, with the increase of street SGR, the decrease in LST of the expressways was the largest, while that of branch roads was the smallest. However, the most significant difference between these four street types was the width. Therefore, the wider the street, the higher the cooling efficiency of plants. In addition, the average R^2^ of four different street types was 0.5181, and the streets’ LST in low-rise and low-density built-up areas with a south-north orientation decreased by 1°C when the SGR increased by 3.57%.

### 4.3. Policy Implications and Limitations

This study used multi-source datasets to explore the relationship between SGR and LST in different street types. Moreover, the street orientation and LCZ concept were also taken into consideration. Based on our results, streets with a south-north orientation, the height of buildings, and the width of a street exert an influence on the street greening effect. Based on the aforementioned results, for the streets in the multi-rise and low-density built-up areas with a south-north orientation, some suggestions were given: (1) Increase the continuity of greening; For these streets with uneven green distribution in the built-up area, new trees can be planted appropriately to build a complete “green corridor”; (2) Enrich the street greening configuration. The suitable combination of arbor, shrubs, and grass can enrich the spatial level of a street’s green belt. The upper layer of the multi-layer structure should be dominated by local tall trees, and the middle and lower layers should be dominated by shrubs and herbs with strong shade resistance. (3) Increase the vertical greening of streets. Vertical greening is also known as three-dimensional greening. Its advantage is that it uses less ground space to obtain more green viewing area, which can improve the environment more. Common vertical greening forms include wall greening, colonnade greening, etc. For the walls of buildings on both sides of the street, the characteristics of climbing plants can be used to achieve green coverage of the building surface during their growth. In addition, planting containers can be installed on the wall surface to achieve green coverage of the wall. (4) The appropriate tree species need to be selected. From the analysis results of the street green sight rate, it can be concluded that the crown width of trees has an essential impact on the street green sight rate. Therefore, within the allowable range of street widths, the green sight rate can be improved by planting trees with a larger crown width and higher leaf density. In tree species selection, local trees with better adaptability to the environment should be the main species, and the proportion of evergreen and deciduous trees should be reasonably allocated.

This study investigated the relationship between streets’ SGR and LST by introducing LCZ. However, there are still some limitations to this study. Firstly, the daytime LST data was acquired to explore its relationship with SGR. However, to further investigate the relationship between streets’ LST and SGR, LST at nighttime should also be used in the consideration of their relationship. Secondly, building data was obtained from Baidu Maps; however, there were differences between the obtained building data and the actual situation. Although building data were calibrated based on Google Earth images, it is difficult to acquire building data with high precision. Finally, the heat produced by human activity in these streets was not taken into consideration, as it was difficult to quantify.

## 5. Conclusions

Relationship exploration between streets’ SGR and LST of different types is of great significance for realizing regional sustainable development goals. Given the lack of consideration of the LCZ concept, Chongqing’s Inner Ring region was used to assess the relationship between the streets’ SGR and LST. First, the LST was retrieved from Landsat 8 imagery after atmospheric correction; next, the SGR of different streets was calculated based on the semantic segmentation method; finally, street types were classified in detail by introducing LCZ, and the relationship between SGR and LST was explored. The results showed that: (1) The LST spatial distribution pattern was closely related to human activity, with the high-temperature zones mainly concentrated in the core commercial areas, dense residential areas, and industrial cluster areas; (2) The average SGR values of expressways, main trunk roads, secondary trunk roads, and branch roads were 21.70%, 22.40%, 24.60%, and 26.70%, respectively. The level of SGR will decrease when the street width increases; (3) There is a negative correlation between the SGR and the LST in most streets. Among them, the LST of secondary trunk roads in low-rise and low-density built-up areas with a south-north orientation had a strong negative correlation with the SGR. Moreover, the wider the street, the higher the cooling efficiency of plants. Specifically, the LST of streets in low-rise and low-density built-up areas with a south-north orientation decreased by 1 °C when the street-greenery rate increased by 3.57%.

## Figures and Tables

**Table 1:** LCZ types of the study area.

Type	Sample
High-rise and high-density built-up area	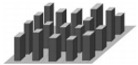
Multi-rise and high-density built-up area	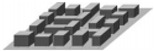
Low-rise and high-density built-up area	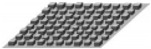
High-rise and low-density built-up area	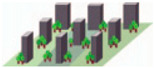
Multi-rise and low-density built-up area	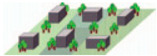
Low-rise and low-density built-up area	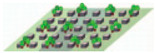
Forest area	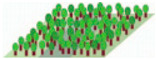
Sand grassland and bare area	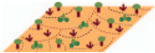
Hard ground area	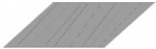
Waterbody area	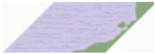

**Table 2:** Range of different block division parameters.

Block Type	Average Building Height/m	Pervious Surface Fraction/%	Building Density/%	Impervious Surface Fraction/%
High-rise and high-density built-up area	≥30	<10	≥20	40–60
Multi-rise and high-density built-up area	10–30	<20	≥20	30–50
Low-rise and high-density built-up area	<10	<30	≥20	20–50
High-rise and low-density built-up area	≥30	30–40	5–20	30–40
Multi-rise and low-density built-up area	10–30	20–40	5–20	30–50
Low-rise and low-density built-up area	<10	30–60	5–20	20–50
Forest area	-	>90	<5	<10
Sand grassland and bare area	-	>90	<5	<10
Hard ground area	-	<10	<5	>90
Waterbody area	-	>90	<5	<10

**Table 3:** The correlation coefficient between the streets’ SGR and LST at different types.

Block Type	Street Type	Street Orientation	Pearson Correlation Coefficient	Sampling Points
High-rise and high-density built-up area	Expressway	South-north	−0.203	68
		East-west	−0.611 **	100
	Main trunk road	South-north	0.003	535
		East-west	−0.085 *	531
	Secondary trunk road	South-north	−0.227 **	489
		East-west	−0.136**	463
	Branch road	South-north	−0.194 **	653
		East-west	−0.060	381
Multi-rise and high-density built-up area	Expressway	South-north	−0.360 **	59
		East-west	−0.067	20
	Main trunk road	South-north	−0.224 **	220
		East-west	0.007	160
	Secondary trunk road	South-north	−0.264 **	153
		East-west	0.022	158
	Branch road	South-north	−0.228 **	201
		East-west	−0.273 **	112
Low-rise and high-density built-up area	Expressway	South-north	/	0
		East-west	/	0
	Main trunk road	South-north	−0.916 **	9
		East-west	/	0
	Secondary trunk road	South-north	−0.813	3
		East-west	/	2
	Branch road	South-north	/	1
		East-west	−0.930	4
High-rise and low-density built-up area	Expressway	South-north	−0.401 **	294
		East-west	−0.174	65
	Main trunk road	South-north	−0.253 **	931
		East-west	−0.167 **	792
	Secondary trunk road	South-north	−0.256 **	578
		East-west	−0.246 **	570
	Branch road	South-north	−0.335 **	431
		East-west	−0.310 **	268
Multi-rise and low-density built-up area	Expressway	South-north	−0.738 **	72
		East-west	−0.174	65
	Main trunk road	South-north	−0.705 **	412
		East-west	−0.092	368
	Secondary trunk road	South-north	−0.709 **	246
		East-west	−0.226 **	207
	Branch road	South-north	−0.727 **	167
		East-west	−0.335 **	112
Low-rise and low-density built-up area	Expressway	South-north	/	0
		East-west	/	0
	Main trunk road	South-north	−0.171	56
		East-west	0.073	23
	Secondary trunk road	South-north	−0.882 **	16
		East-west	0.673 **	14
	Branch road	South-north	−0.129	18
		East-west	−0.664 **	16
Forest area	Expressway	South-north	−0.620 *	16
		East-west	−0.476 *	19
	Main trunk road	South-north	−0.165*	151
		East-west	0.186	64
	Secondary trunk road	South-north	−0.245	62
		East-west	−0.356 **	52
	Branch road	South-north	−0.104	46
		East-west	−0.493 *	22
Sand grassland and bare area	Expressway	South-north	0.505 *	16
		East-west	0.840	3
	Main trunk road	South-north	0.023	107
		East-west	−0.008	69
	Secondary trunk road	South-north	0.041	61
		East-west	−0.792 **	25
	Branch road	South-north	0.010	25
		East-west	−0.349	16
Hard ground area	Expressway	South-north	−0.326 **	122
		East-west	−0.478 **	104
	Main trunk road	South-north	−0.183 **	315
		East-west	−0.054	165
	Secondary trunk road	South-north	−0.484 **	93
		East-west	−0.474 **	94
	Branch road	South-north	−0.173	77
		East-west	−0.325	36

Note: * shows that the Pearson correlation coefficient of this type of street has passed the significance test of 95% confidence level; ** shows that the Pearson correlation coefficient of this type of street has passed the significance test of 99% confidence level.

## Data Availability

The data presented in this study are available on request from the corresponding author.

## Abbreviations

LCZ	Local climate zone	A term for describing regional local climate
LST	Land surface temperature	An index for describing regional surface temperature
SGR	Street-greenery rate	An index for describing the street greenery rate
